# Incidence of chronic obstructive pulmonary disease based on three spirometric diagnostic criteria in Sao Paulo, Brazil: a nine-year follow-up since the PLATINO prevalence study: Incidência de doença pulmonar obstrutiva crônica com base em três critérios diagnósticos espirométricos em São Paulo, Brasil: seguimento de nove anos após o estudo de prevalência PLATINO

**DOI:** 10.1590/1516-3180.2015.9620902

**Published:** 2014-12-19

**Authors:** Graciane Laender Moreira, Mariana Rodrigues Gazzotti, Beatriz Martins Manzano, Oliver Nascimento, Rogelio Perez-Padilla, Ana Maria Baptista Menezes, José Roberto Jardim

**Affiliations:** I PhD. Physiotherapist, Department of Translational Medicine, Universidade Federal de São Paulo (Unifesp), São Paulo, Brazil.; II PhD. Professor, Department of Physiotherapy, Centro Universitário São Camilo, São Paulo, Brazil.; III MD, PhD. Pulmonology Sector, Universidade Federal de São Paulo (Unifesp), São Paulo, Brazil.; IV MD. Adjunct Professor, National Institute of Respiratory Diseases, Mexico City, Mexico.; V MD, PhD. Adjunct Professor, Department of Internal Medicine, School of Medicine, Universidade Federal de Pelotas (UFPel), Pelotas, Rio Grande do Sul, Brazil.; VI MD, PhD. Adjunct Professor, Universidade Federal de São Paulo (Unifesp), São Paulo, Brazil.

**Keywords:** Pulmonary disease, chronic obstructive, Incidence, Diagnosis, Epidemiology, Spirometry, Doença pulmonar obstrutiva crônica, Incidência, Diagnóstico, Epidemiologia, Espirometria

## Abstract

**CONTEXT AND OBJECTIVE:**

Chronic obstructive pulmonary disease (COPD) is a respiratory disease of high prevalence and socioeconomic impact worldwide. It affects approximately 16% of the population of São Paulo. The incidence of COPD is still unknown in Brazil. The aim of this study was to estimate new cases of COPD in a population-based sample in São Paulo, Brazil, using three different spirometric diagnostic criteria, and to assess the concordance between these criteria.

**DESIGN AND SETTING:**

Prospective cohort study, in the city of São Paulo, Brazil.

**METHODS:**

A questionnaire was applied and anthropometry and pre and post-bronchodilator spirometry were performed on the same subjects as in the initial PLATINO study (2003) in São Paulo. Data from this follow-up study were added to the original database of the initial phase. Incident COPD cases refer to subjects who developed the disease in accordance with each spirometric criterion during the nine-year follow-up period. The Statistical Package for the Social Sciences, version 17.0 (SPSS Inc., Chicago, IL, USA) was used in the analysis and the significance level was set at P < 0.05.

**RESULTS:**

613 subjects participated in the follow-up. New COPD cases ranged in frequency from 1.4% to 4.0%, depending on the diagnostic criterion used. The concordance between the criteria ranged from 35% to 60%.

**CONCLUSION:**

The incidence of COPD after a nine-year follow-up was high, but varied according to the spirometric criterion used. The agreement between the criteria for identifying new cases of the disease ranged from 35% to 60%.

## RESUMO

**CONTEXTO E OBJETIVO:**

A doença pulmonar obstrutiva crônica (DPOC) é uma enfermidade respiratória de alta prevalência e grande impacto socioeconômico no mundo e afeta aproximadamente 16% da população paulista. A incidência da DPOC ainda é desconhecida no Brasil. O objetivo deste estudo foi avaliar os novos casos de DPOC numa amostra de base populacional em São Paulo, Brasil, considerando três diferentes critérios diagnósticos espirométricos, e avaliar a concordância entre esses critérios.

**TIPO DE ESTUDO E LOCAL:**

Estudo de coorte prospectivo, na cidade de São Paulo, Brasil.

**MÉTODOS:**

Foi aplicado um questionário e realizadas a antropometria e espirometria pré e pós-broncodilatador nos mesmos indivíduos do Estudo PLATINO inicial (2003), de São Paulo. Dados deste estudo de seguimento foram adicionados ao banco de dados original da fase inicial. Os casos incidentes da DPOC se referem aos indivíduos que desenvolveram a doença de acordo com cada critério espirométrico durante os nove anos de seguimento. Foi utilizado o programa Statistical Package for the Social Sciences, version 17.0 (SPSS Inc., Chicago, IL, USA) na análise e o nível de significância foi P < 0.05.

**RESULTADOS:**

Participaram do seguimento 613 indivíduos. Os novos casos de DPOC variaram de 1,4% a 4,0%, dependendo do critério diagnóstico utilizado, e a concordância entre os critérios para identificar os casos incidentes variou de 35% a 60%.

**CONCLUSÃO:**

A incidência da DPOC após nove anos de seguimento foi alta, porém variável de acordo com o critério espirométrico utilizado. A concordância entre os critérios para identificar os novos casos da doença variou de 35% a 60%.

## INTRODUCTION

Chronic obstructive pulmonary disease (COPD) is a respiratory disease that has had great socioeconomic impact due to its high prevalence worldwide.^1^ The prevalence of COPD in a population aged 40 years and older in São Paulo was found to be 15.8%, through the PLATINO study.^2^ Epidemiological studies in European, Asian and North American countries have estimated the prevalence of COPD in their populations to be 5-25%,^3^ with incidence rates ranging from 1.5-11%.^4^^,^^5^^,^^6^^,^^7^^,^^8^^,^^9^^,^^10^^,^^11^ This variability can be attributed to the different spirometry criteria used to diagnose the disease.^3^

Among the spirometry criteria used in diagnosing airflow obstruction, the ones most commonly used are a fixed ratio of less than 0.7 between forced expiratory volume in one second (FEV_1_) and forced vital capacity (FVC) (FEV_1_/FVC < 0.7)^12^ and a FEV_1_/FVC ratio below the lower limit of normality (LLN),^13^ which varies according to the subjects’ age, height, race and sex, as obtained using reference equations. However, it has been suggested that using forced expiratory volume in six seconds (FEV_6_) would be an alternative to FVC, since this is approximately 25% less variable than FVC^14^ and is a good substitute for detecting airflow obstruction.

Measuring and monitoring a public health problem is the first step in elaborating public health administrative strategies.^15^ Spirometry is essential in diagnosing COPD,^16^ regardless of the spirometric criteria used to identify airflow obstruction. Therefore, this follow-up study, which includes the subjects who took part in the initial phase of the PLATINO study,^2^ in São Paulo, is quite important, given that new cases of COPD can be identified using the same diagnostic procedures and that differences in the estimated incidence can be found in terms of the different spirometric criteria.

## OBJECTIVE

The aim of the present study was to evaluate new cases of COPD at the end of a nine-year follow-up period, analyzing the same subjects who took part in the initial phase of the population-based PLATINO study in the city of São Paulo and taking into consideration three different diagnostic criteria: FEV_1_/FVC < 0.70 post-bronchodilator; FEV_1_/FVC < LLN post-bronchodilator; and FEV_1_/FEV_6_ < LLN post-bronchodilator; and to identify the concordance of these criteria in the new cases.

## METHODS

### Sample and study design

The present study population was composed of the same subjects who took part in the initial phase of the population-based PLATINO study in São Paulo in 2003 (n = 1,000), in order to evaluate the prevalence of COPD in this city.

The initial PLATINO study (2003) began with stratification of the metropolitan area into the main municipality and its suburbs. These regions were then divided into socioeconomic segments. Taking into consideration the stratification and a sampling probability proportional to the size and the number of houses, 68 census tracts were selected from each segment (42 in the city of São Paulo and 26 in the other municipalities). The number of individuals within each census tract was updated based on the last census at the location. An average of 15 households (selected from the National Household Sampling Survey, 2002) was visited and systematic sampling was obtained.

A map of each census tract was drawn, and the blocks (or similar units) were numbered. In each sector, one block was randomly selected and one corner of the block was chosen. From the corner selected, every second house was visited until the necessary number of homes had been obtained. Data on the people living in each house were gathered and recorded in order to obtain the distribution according to age and sex. All the adults aged 40 years or older living in the selected households were invited to take part in the study, and they were included in the survey. The sample was self-weighted in each municipality.

The sample size calculations indicated that 800 individuals would be needed in each center if the prevalence was assumed to be up to 30% with a margin of error of less than 4 percentage points. The objective was to interview 20% more subjects per center, with an identical percentage of losses and refusals to participate. The final sample of the initial phase of the PLATINO study in São Paulo included 1,000 subjects.

All of the houses in which the subjects were interviewed in the initial phase of the PLATINO study were visited again in 2012. The initial contact was made by one of four screeners (the addresses were obtained from the evaluation forms of the initial phase of the PLATINO study), who confirmed whether the subjects still lived at those addresses, checked their telephone numbers and informed them about the interviewers’ intended visit.

If the subjects no longer lived in the same house, the screeners sought to determine their whereabouts through information from neighbors and inquiries in neighborhood businesses, or by consulting telephone directories. The screeners visited subjects who had moved to another house in the same area. Subjects who had moved to another city within the state of São Paulo were contacted by phone and visited in their new city to determine whether they wished to continue taking part in the study. The names of subjects whose whereabouts were unknown were looked up in the registry of deaths within the state of São Paulo and other Brazilian states. All data obtained by the screeners were sent to the coordinating body responsible for organizing the files for the subsequent scheduling of interviews.

At each respondent’s house, the researchers first asked for written informed consent from the subject. If the subject agreed to participate in the study, they proceeded to gather data in accordance with the following sequence: anthropometric assessment for body mass index (BMI) calculation; completion of the questionnaire with exclusion criteria for participation in spirometry; pre-bronchodilator spirometry; administration of a portion of the main questionnaire (during the first 15 minutes after bronchodilator administration); post-bronchodilator spirometry; and administration of the remainder of the main questionnaire.

The main questionnaire (PLATINO questionnaire) was composed of questions relating to demographic and social variables, reports of respiratory symptoms, medication information, diagnoses of respiratory diseases and comorbidities, smoking history, anti-influenza vaccinations, quality of life (SF-12 quality of life questionnaire) and indoor pollution and dust exposure.

### Diagnosis

The diagnosis of COPD was confirmed by means of spirometry, using a portable battery-powered spirometer and an ultrasound system (Easy One spirometer; NDD Medical Technologies, Andover, MA, USA, and Zurich, Switzerland) that was identical to those used in the initial phase of the PLATINO study. The subjects performed up to 15 forced expiratory maneuvers (which was the maximum accepted by the team in one session), in order to obtain three acceptable maneuvers yielding the highest FEV_1_ and FVC values without exceeding a difference of 150 ml. Subsequently, an inhaled bronchodilator (salbutamol, 200 µg) was administered using a 500-ml spacer. The test was then repeated 15 minutes later. All spirometric tests were performed with the subjects seated and using a nose clip and a disposable mouthpiece. Only the expiratory phase was recorded.

In both the initial and the follow-up (2012) phase, the subjects were diagnosed using three criteria: FEV_1_/FVC < 0.70 post-bronchodilator;^12^ FEV_1_/FVC < LLN post-bronchodilator;^13^ and FEV_1_/FEV_6_ < LLN post-bronchodilator.^17^

The reference equation derived from the PLATINO study was used to calculate the predicted values and the LLN for FEV_1_, FEV_6_, FVC, FEV_1_/FVC, and FEV_1_/FEV_6_.^18^

The present study was approved by the university’s Research Ethics Committee (no. 04234/10). After being informed about the study and its procedures, all the subjects who agreed to participate provided written informed consent.

### Statistical analysis

The data gathered during the follow-up phase of the PLATINO study were added to the original database of the initial phase of the study, which had been conducted in the city of São Paulo, Brazil, in 2003. The data analysis was performed using the Statistical Package for the Social Sciences, version 17.0 (SPSS Inc., Chicago, IL, USA), and the statistical significance level was set at P < 0.05.

Cumulative incidence refers to the number of onsets in a population at risk during a certain period. In this study, the new cases of COPD were obtained from among the subjects who developed the disease, in accordance with each spirometric diagnostic criterion, over the nine-year follow-up period. Preexisting cases, individuals who did not take part in the follow-up phase and individuals who did not undergo spirometry in either the initial or the follow-up phase of the PLATINO study were not included in the incidence calculation. Subjects who reported having a medical diagnosis of asthma and cases of FEV_1_ reversibility, as seen in the post-bronchodilator spirometry were considered eligible for analysis.

## RESULTS

**Figure 1.**
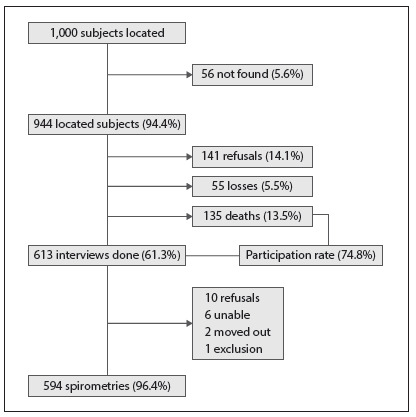
Flowchart for final sample in follow-up PLATINO study, São Paulo, Brazil.

Out of the 1,000 subjects who took part in the initial phase of the PLATINO study, 944 were found again or information on their whereabouts after nine years was obtained. The reasons for the loss of follow-up were as follows: subject not found (n = 56), death (n = 135), refusal to participate (n = 141) and subject moved to another state/city and considered to be a “loss” (n = 55). Out of these 944 subjects, 613 took part in the follow-up phase of the study and 594 provided consent and underwent pre-bronchodilator and post-bronchodilator spirometry. The participants and non-participants (i.e. refusals, subject not found and subject who had moved to another state/city) of the follow-up PLATINO study had the same characteristics as the participants who took part in the initial phase of the study, in terms of sex, age, education level and pulmonary function. The only significant difference was in the proportion of former smokers, which was higher in the group of subjects who took part in the follow-up phase of the study, in relation to non-participants (37.6% versus 23.8%; P < 0.001). The data regarding the subjects who took part in the follow-up PLATINO study in São Paulo are presented in Figure 1.

The demographic and clinical data on the subjects who took part in the initial phase (n = 1,000) and follow-up phase (n = 613) of the PLATINO study are presented in Table 1. At the end of the nine-year follow-up period, the subjects showed deterioration in the physical domain of quality of life. The proportion of smokers was different, and thus, the proportion of former smokers was higher.

**Table 1.**
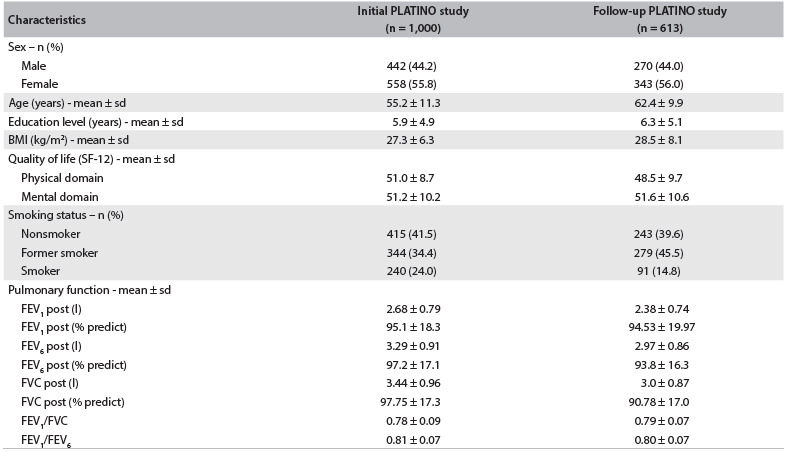
Clinical, anthropometric and functional characteristics of initial PLATINO participants (n = 1,000) and follow-up PLATINO participants (n = 613)

FEV_1_ = forced expiratory volume in first second; FVC = forced vital capacity; FEV_6_ = forced expiratory volume in six seconds; BMI = body mass index; sd = standard deviation.

**Figure 2.**
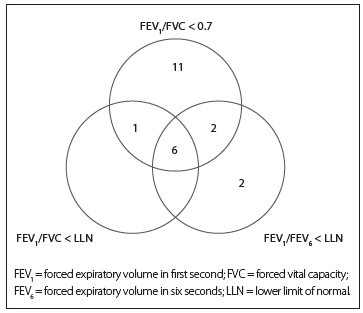
Incident cases of chronic obstructive pulmonary disease (COPD) and agreement among three spirometric diagnostic criteria: FEV_1_/FVC < 0.7 post-bronchodilator; FEV_1_/FVC < LLN post-bronchodilator; and FEV_1_/FEV_6_ < LLN post-bronchodilator.

The new cases of COPD at the end of the nine-year follow-up period varied in terms of the spirometric criterion used to diagnose the disease: FEV_1_/FVC < 0.7 showed the highest proportion of onsets (Table 2). The three diagnostic criteria were concordant with regard to classifying six subjects who had COPD (Figure 2). The criterion FEV_1_/FEV_6_ < LLN with FEV_1_/FVC < LLN presented the highest level of concordance (60.0%) in classifying new COPD cases, followed by FEV_1_/FVC < 0.7 with FEV_1_/FEV_6_ < LLN (40.0%), and lastly, FEV_1_/FVC < 0.7 with FEV_1_/FVC < LLN (35.0%). The results regarding smoking status among the incident cases for the three criteria were as follows: GOLD criterion^12^ (40% nonsmokers, 35% smokers and 25% former smokers); FEV_1_/FVC < LLN (43% nonsmokers, 43% smokers and 14% former smokers); and FEV_1_/FEV_6_ < LLN (23% nonsmokers, 44% smokers and 33% former smokers).

**Table 2.**
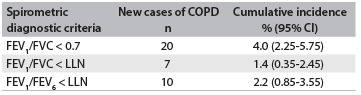
Incident cases of chronic obstructive pulmonary disease (COPD) in São Paulo, Brazil, based on three different spirometric diagnostic criteria

FEV_1_ = forced expiratory volume in first second; FVC = forced vital capacity; FEV_6_ = forced expiratory volume in six seconds; LLN = lower limit of normal; 95% CI = 95% confidence interval.

## DISCUSSION

The results from this study showed that, at the end of a nine-year follow-up period, the rate of diagnosing new cases of COPD in a population-based sample in the city of São Paulo ranged from 1.4% to 4.0%, depending on the spirometric diagnostic criterion used. The guidelines available regarding COPD diagnosis and treatment^12^^,^^19^^,^^20^^,^^21^ differ in relation to the spirometric criteria used for identifying airflow obstruction, and there is controversy regarding which criterion is the most appropriate for diagnosing the disease.

The spirometric diagnostic criterion that takes a fixed FEV_1_/FVC ratio or a FEV_1_/FVC of 0.7 is simple and practical. However, it can increase the rates of false-positive results among older subjects and might not detect the disease in younger subjects (false-negative results).^22^^,^^23^ The increase in the number of false-positive results is minimized when this criterion is based on the fifth percentile or taken as below the LLN. On the other hand, it has already been demonstrated that false-negative results are more common when such a criterion is used. Because of the resulting misdiagnoses, COPD patients are undertreated or even not treated in the initial stages of the disease, when they could benefit from treatment.^24^ The criterion FEV_1_/FEV_6_ < 0.75 or FEV_1_/FEV_6_ < LLN has the advantages of being easy to apply, both for the evaluator and for the patient. This criterion can eliminate the limitation on low-flow accuracy at the end of the FVC maneuver and can reduce the time needed to perform the spirometry, as well as the chances of possible complications during the examination, such as syncope.^25^^,^^26^^,^^27^ These diagnostic criteria present different sensitivities, specificities and positive and negative predictive values.^23^^,^^24^^,^^28^^,^^29^

Although the positive and negative points of each COPD diagnostic criterion have been identified, the historical divergences regarding the use of fixed ratios, LLN, and other criteria still remain in the literature.^30^^,^^31^ Therefore, further studies that aim to identify the diagnostic and prognostic markers of this disease are needed.

Hence, the present study evaluated the findings from new COPD cases in the city of São Paulo based on three different spirometric criteria. The incidence rate of COPD was lower (n = 7) when FEV_1_/FVC < LLN was used, followed by FEV_1_/FEV_6_ < LLN (n = 10) and the fixed-ratio criterion FEV_1_/FVC < 0.7 (n = 20). Thus, according to the diagnostic criterion used, the number of new COPD cases may triple. This difference in the results was also reported previously in an incidence study^11^ in which the results indicated that the number of cases could double. This large disparity probably reflects differences in sensitivity and specificity.^32^ In divergent cases of airflow obstruction (e.g. FEV_1_/FVC < 0.7 and FEV_1_/FVC ≥ LLN), the subjects would not have clinically significant obstruction but would have a clinical profile characterized by significant comorbidities, thereby indicating that they could be at risk of developing COPD and should be followed up carefully.^33^

Despite the disparities among the criteria, six subjects showed concordance regarding the incidence of airflow obstruction. The concordance between the criteria FEV_1_/FVC < 0.7 and FEV_1_/FVC < LLN was 35.0%, while de Marco et al*.*^*11*^ reported concordance of 48% for these same criteria. The most discordant cases were associated with the criterion FEV_1_/FVC < 0.7, which was the only one to classify 11 subjects as new cases.

From investigating the characteristics of these 11 subjects in relation to the COPD cases with concordance in three criteria (n = 6), we found that 64% were 68 years of age or older (P = 0.04). Considering that aging contributes to airflow limitation, mainly due to increased thoracic wall rigidity and decreased pulmonary elastic recoil,^34^^,^^35^ these findings corroborate the assumption that the criterion FEV_1_/FVC < 0.7 may lead to misdiagnosing of COPD among the elderly through precluding the clinical distinction between disease and airflow limitation that is caused by the aging process.^13^^,^^36^ Mannino et al*.*^*37*^ confirmed that there is uncertainty regarding whether discordant cases obtained through using different criteria represent misdiagnosis, COPD phenotypes or overlapping between COPD and any other associated disease.

The use of different criteria can lead to variability in diagnosing new COPD cases, which can affect public health decisions regarding strategic planning, allocation of resources and establishment of priorities.^22^ A study published in 2013^17^ demonstrated that using the FEV_1_/FEV_6_ criterion was more reliable than using FEV_1_/FVC, given that FVC varies with expiratory time during the forced maneuvers required for spirometry. The present study also suggests that FEV_1_/FEV_6_ is the most appropriate criterion, considering its greater concordance with the other two spirometric diagnostic criteria (FEV_1_/FVC < 0.7 and FEV_1_/FVC < LLN), which produced extreme numbers of new COPD cases, i.e. a higher and a lower proportion of cases, respectively. Thus, further studies are needed, especially longitudinal population-based studies, in order to identify the best diagnostic criterion to use.

When considering the use of different criteria to evaluate the diagnoses of new cases of COPD, care should be taken in comparing the rate found in the present study with the rates obtained in other studies. This is because, in addition to the criterion used for diagnosing the disease,^38^ there were differences in incidence rate,^9^^,^^10^^,^^11^^,^^38^ follow-up duration,^5^^,^^7^^,^^8^^,^^38^ population studied,^5^^,^^8^^,^^10^ age distribution^9^^,^^11^ and exclusion criteria.^7^^,^^9^^,^^10^^,^^11^ This high variability in identifying new cases of COPD among different studies may be associated with the fact that the samples of some of the studies did not include subjects who reported asthma,^9^^,^^11^ as well as differences in follow-up periods, which ranged from 7 to 25 years.

One of the limitations of the present study is the rate of losses from the follow-up, which exceeded 20%. However, a European multicenter longitudinal study over a similar follow-up period reported a participation rate of 63.3%.^11^ In addition, the group formed by the cases of loss from the follow-up and refusal to participate had the same clinical and pulmonary function characteristics as shown by the participants in the follow-up phase of the PLATINO study. Another limitation is the fact that there was only one re-evaluation after a period of nine years, which does not allow the incidence rates to be expressed as numbers of cases/year, although the perceptual value could be presented and used in comparisons with other studies.

## CONCLUSION

We conclude that the rate of diagnosing new cases of COPD in the city of São Paulo at the end of a nine-year follow-up period was similar to the percentages found in European, Asian and North American countries, with a range from 1.4% to 4.0%, depending on the spirometric criterion used for diagnosing the disease, with concordance levels of 35-60%. Therefore, researchers should concentrate their efforts on determining which spirometric criterion is the most accurate one for diagnosing COPD, so that epidemiological studies can better report the impact of this disease and the real responses of subjects to the treatment prescribed.
